# Supplementary material for: Recycling polyolefin plastic waste at short contact times via rapid joule heating
Source: Nat Commun. 2024 Jul 5;15:5662. doi: 10.1038/s41467-024-50035-3 (PMC11226686; doi:10.1038/s41467-024-50035-3)
Supplement: Supplementary file 1 — Supplementary Information [file 41467_2024_50035_MOESM1_ESM.pdf]

# Supplementary Information for

**Recycling Polyolefin Plastic Waste at Short Contact Times via Rapid Joule Heating**

Esun Selvam[1,2†], Kewei Yu[2†], Jacqueline Ngu[1,2], Sean Najmi[2,3], Dionisios G. Vlachos[1,2,3]*

[1]Center for Plastics Innovation, University of Delaware, 221 Academy St., Newark, DE, 19716, USA

[2]Department of Chemical and Biomolecular Engineering, University of Delaware, 150 Academy St, Newark, DE, 19716, USA

[3]Delaware Energy Institute, University of Delaware, 221 Academy St., Newark, DE, 19716, USA

[†]These authors contributed equally.

*Corresponding author. Email: vlachos@udel.edu

**This file includes:**

Supplementary Figures 1-22

Supplementary Tables 1 and 2

References

**Supplementary Figures and Tables**

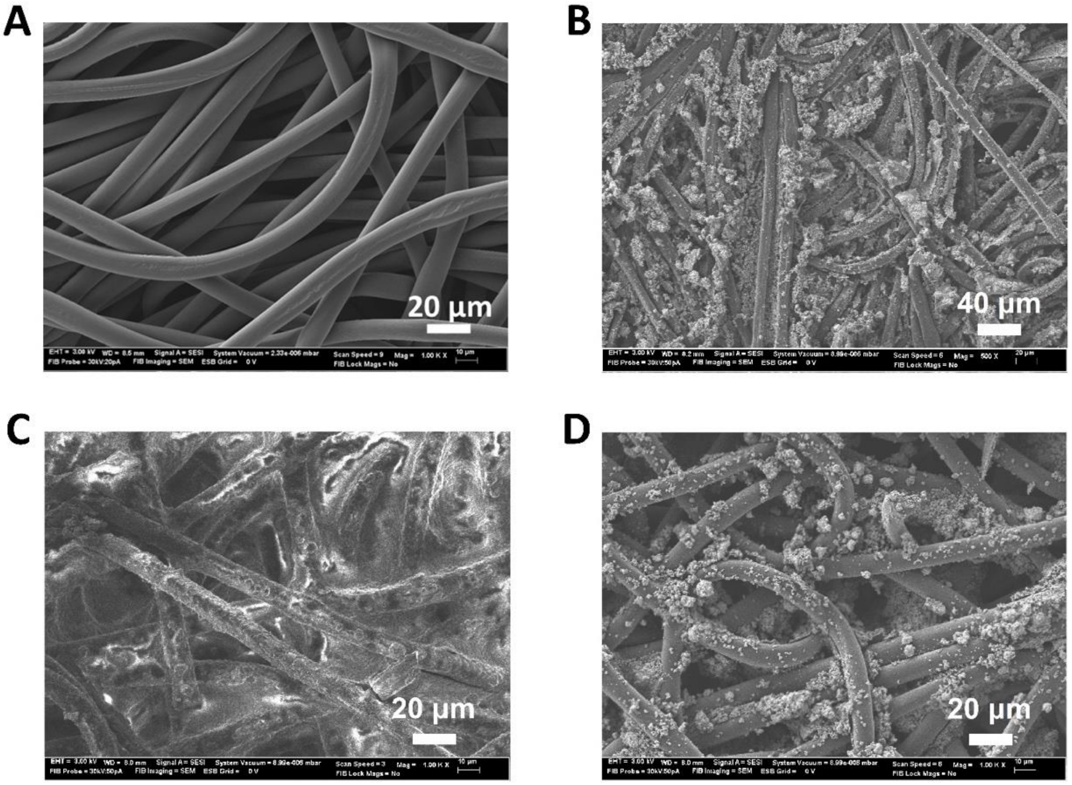


**Supplementary Figure 1.** SEM Images of (A) Fresh carbon fiber paper (1K X Mag) (CFP), (B) CFP coated with H-ZSM-5 (500 X Mag), (C) CFP coated with H-ZSM-5 and LDPE (1K X Mag), (D) Post-reaction CFP after RPH of LDPE over H-ZSM-5 (1K X Mag).

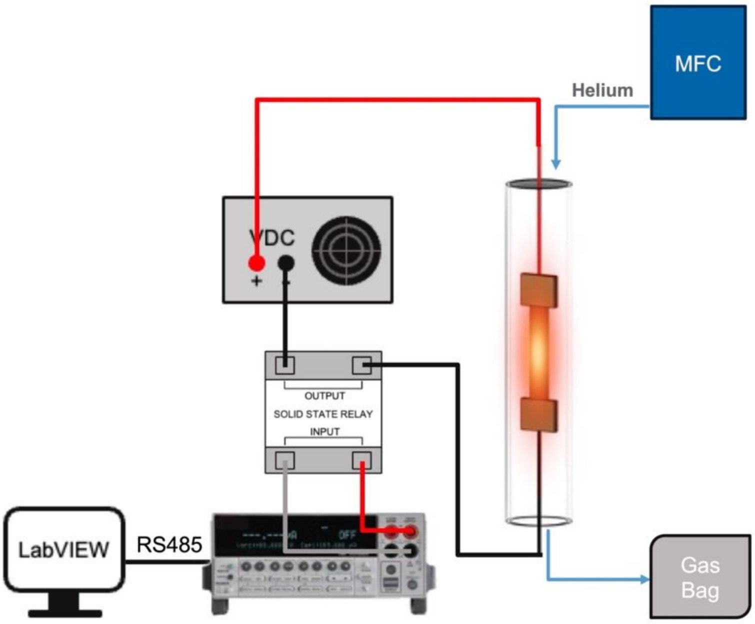


**Supplementary Figure 2.** Schematic of experimental setup for Joule heating reactor

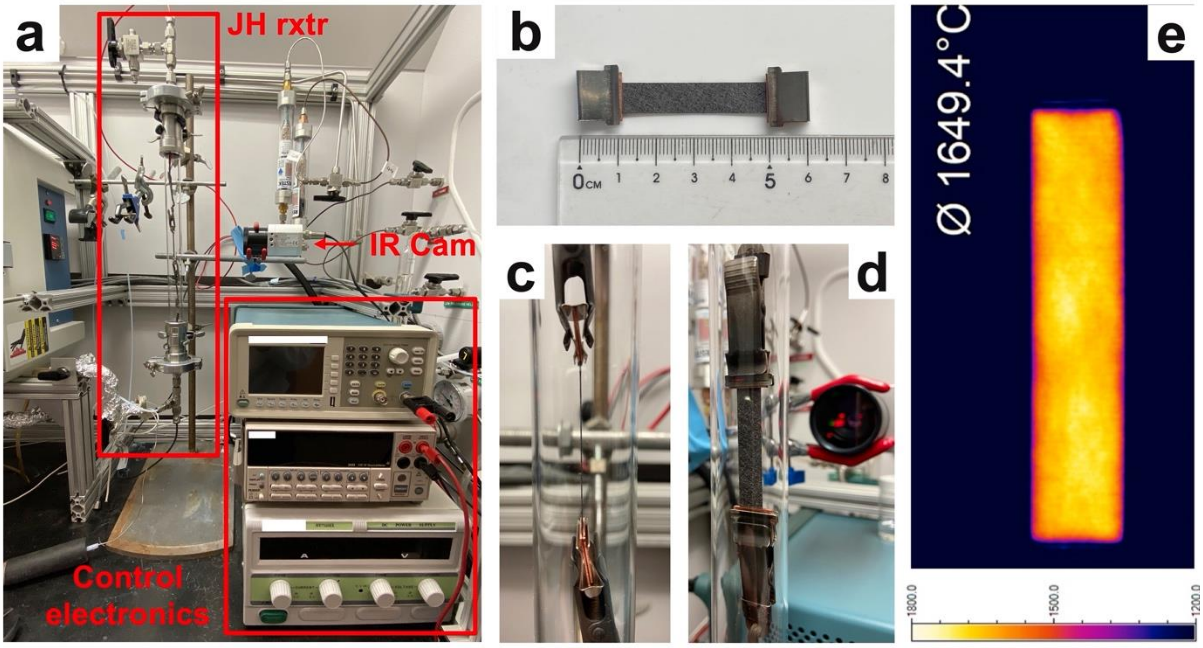


**Supplementary Figure 3.** (a) Experimental setup for Joule heating reactor. (b) CFP - heating element. (c-d) Zoomed-in snapshot of heating element inside reactor and position of IR camera (e) Sample temperature profile of CFP obtained using an IR camera.

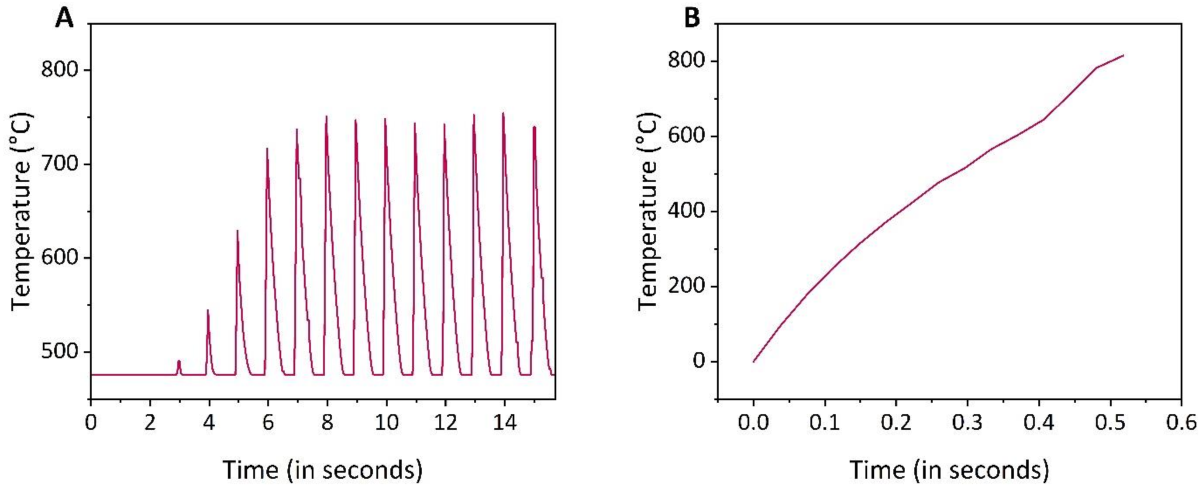


**Supplementary Figure 4.** Typical temperature profile for (A) Rapid Pulse Heating and (B) Continuous Joule heating of LDPE.

For CJH, the temperature profiles were extrapolated to 0 °C since the IR Camera can only detect temperatures between 450 °C to 1800 °C.

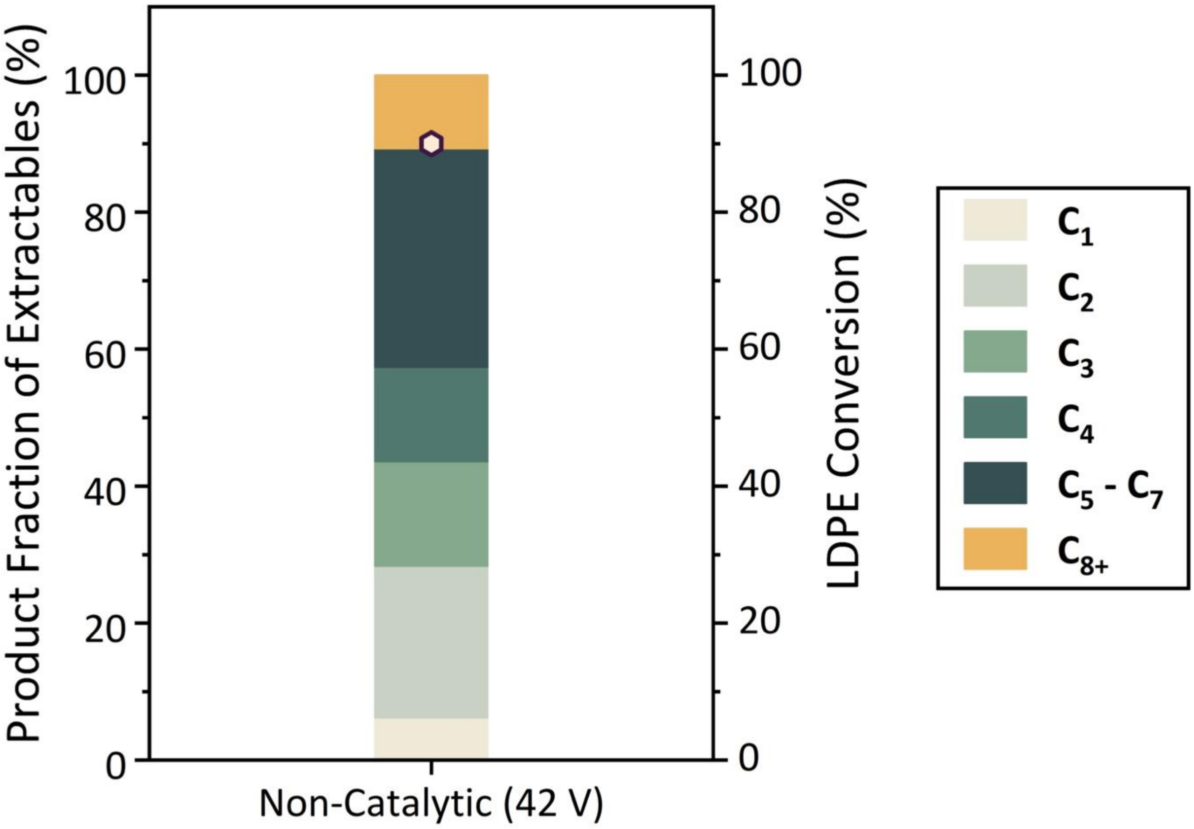


**Supplementary Figure 5.** Product selectivity (left) and conversion (right) from non-catalytic RPH of LDPE obtained using GC-FID.

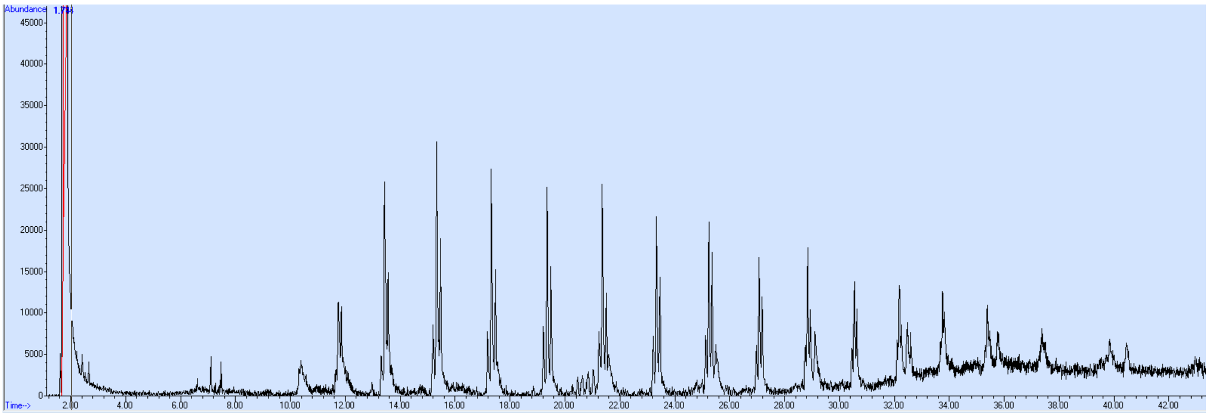


**Supplementary Figure 6.** Product distribution of alkanes and olefin liquid products in non-catalytic RPH of LDPE obtained using GC-MS.

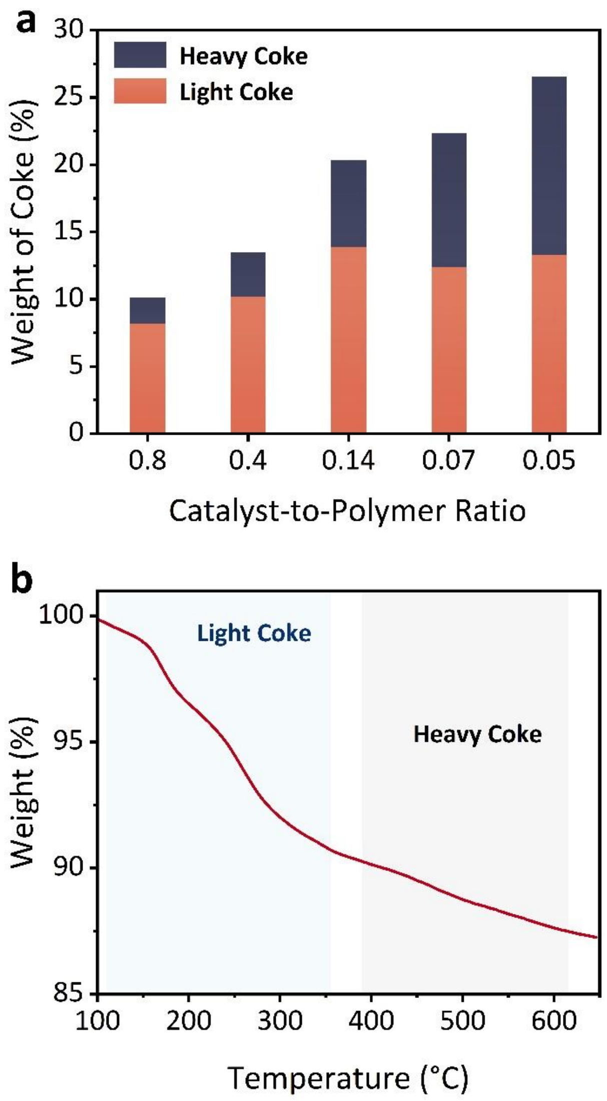


**Supplementary Figure 7.** (a) Wt loss (%) of coke on spent catalysts at different catalyst-to-polymer ratios (42 V and 10 pulses) from TGA. (b) An example of TGA weight loss data for spent catalyst obtained from a reaction performed at a catalyst-to-polymer ratio of 0.4.

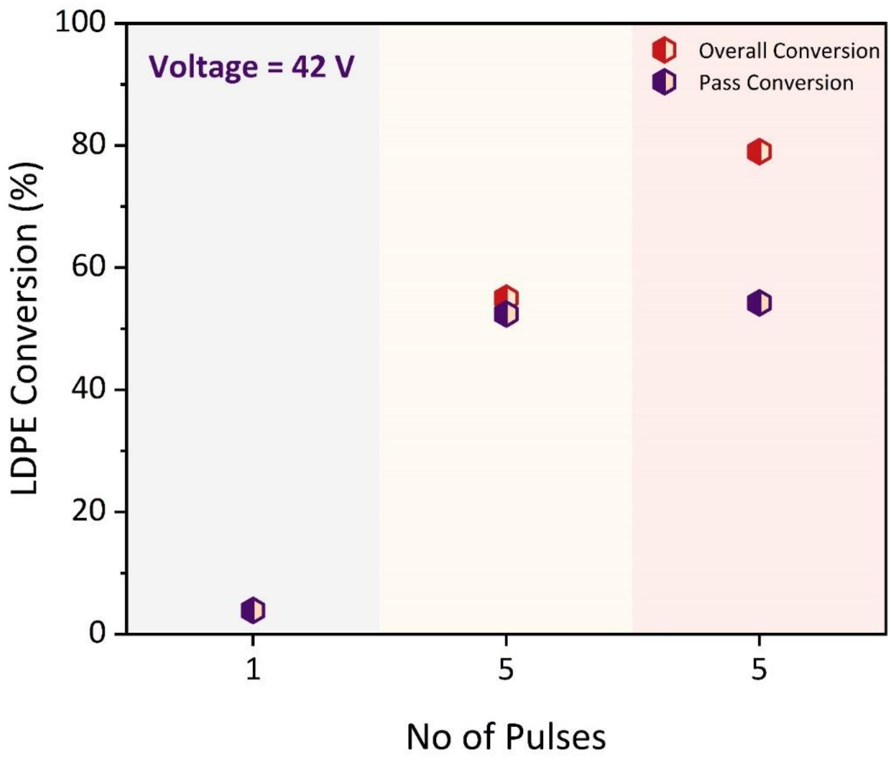


**Supplementary Figure 8.** Performance of a sequential pulsing of two 5 pulse sequences for RPH of LDPE over H-ZSM-5 at 42 V.

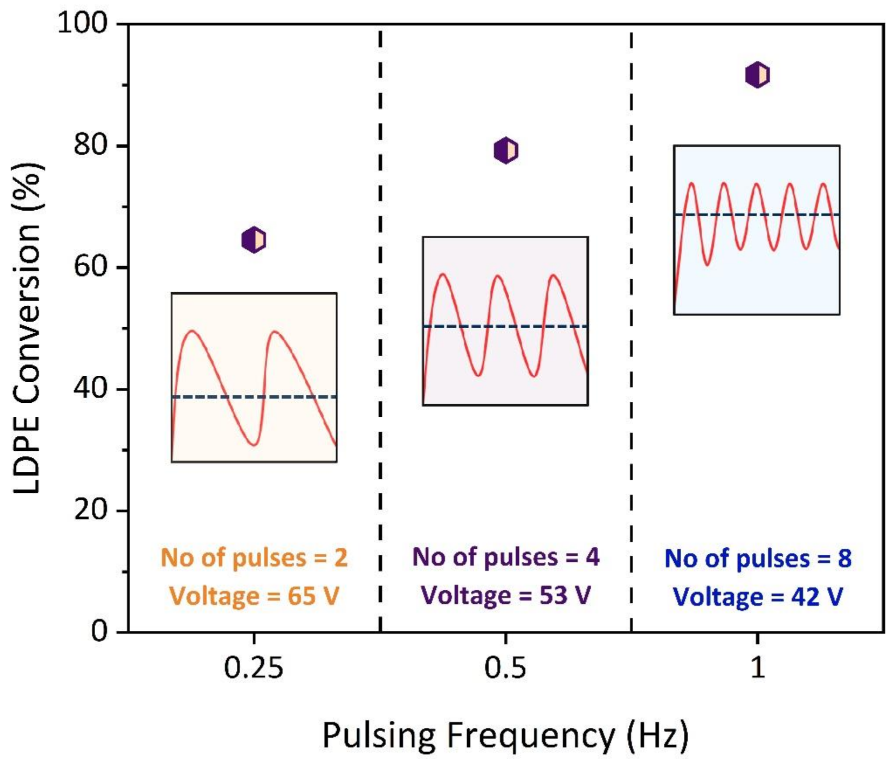


**Supplementary Figure 9.** Effect of pulsing frequency (controlled by varying cooling times) whilst maintaining the peak temperatures (Tmax ~720 °C) and the total exposure time (8 seconds) constant.

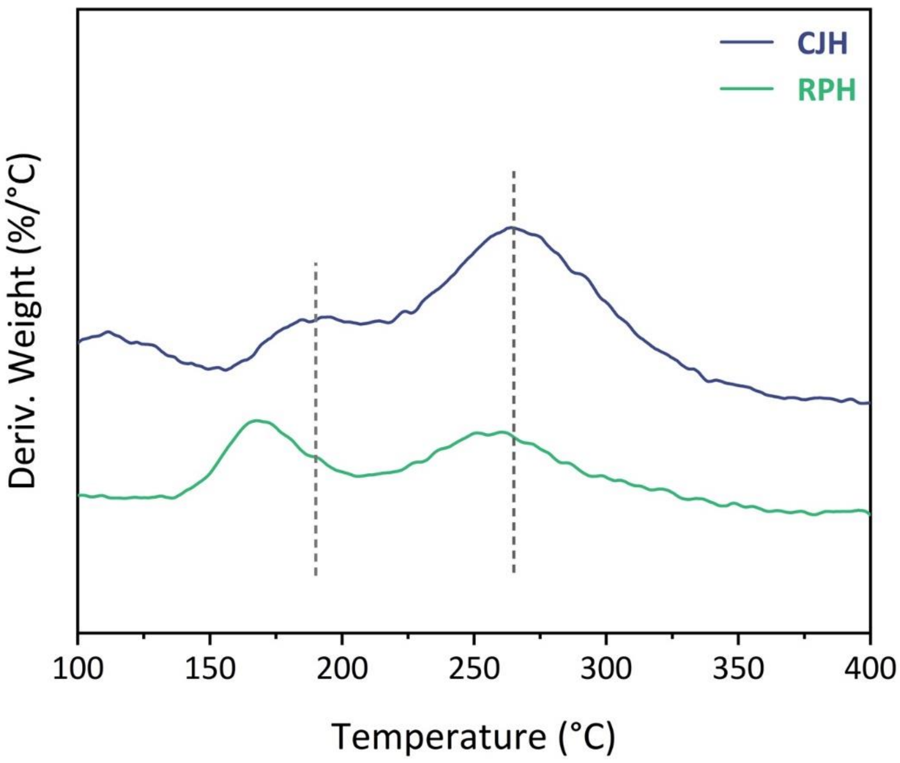


**Supplementary Figure 10.** Comparison of DTA curves of spent catalysts from RPH and CJH after 3 cycles of reuse.

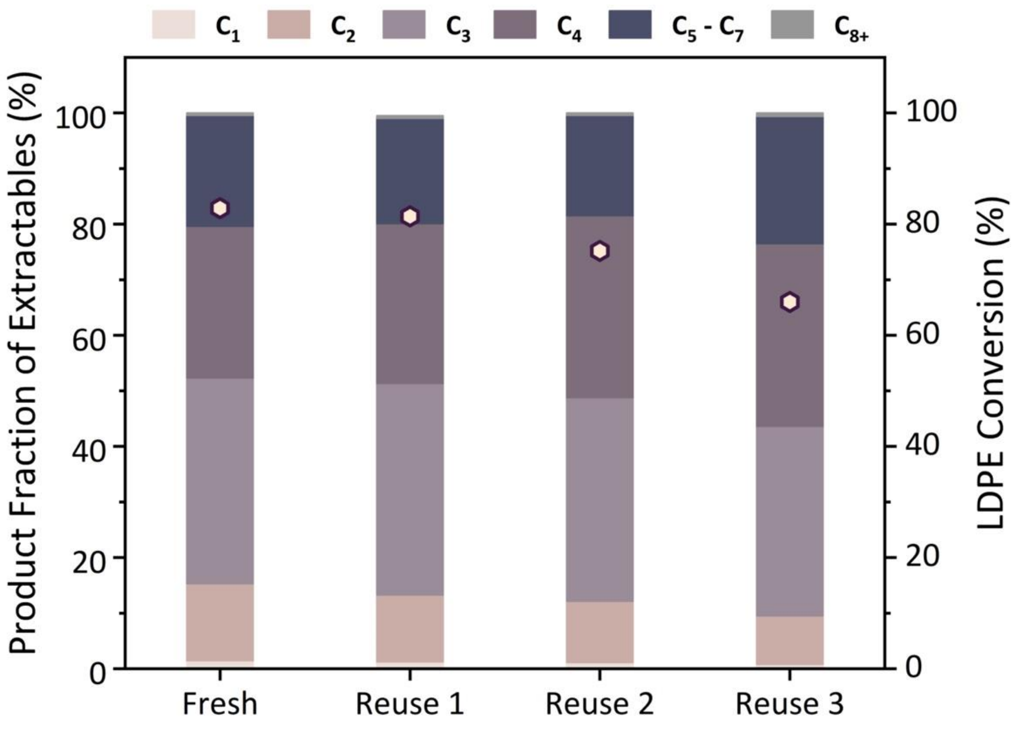


**Supplementary Figure 11.** Reusability of CFP coated with H-ZSM-5 for CJH of LDPE (26 V and 500 ms) with steam co-feeding.

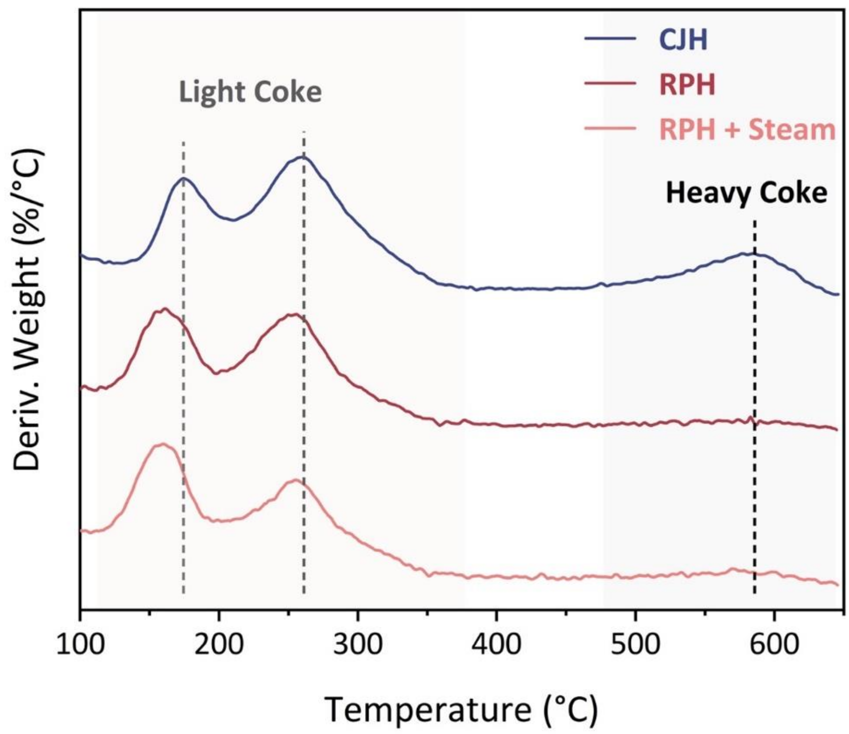


**Supplementary Figure 12.** Comparison of DTA curves of spent catalysts from RPH with and without co-feeding steam and CJH.

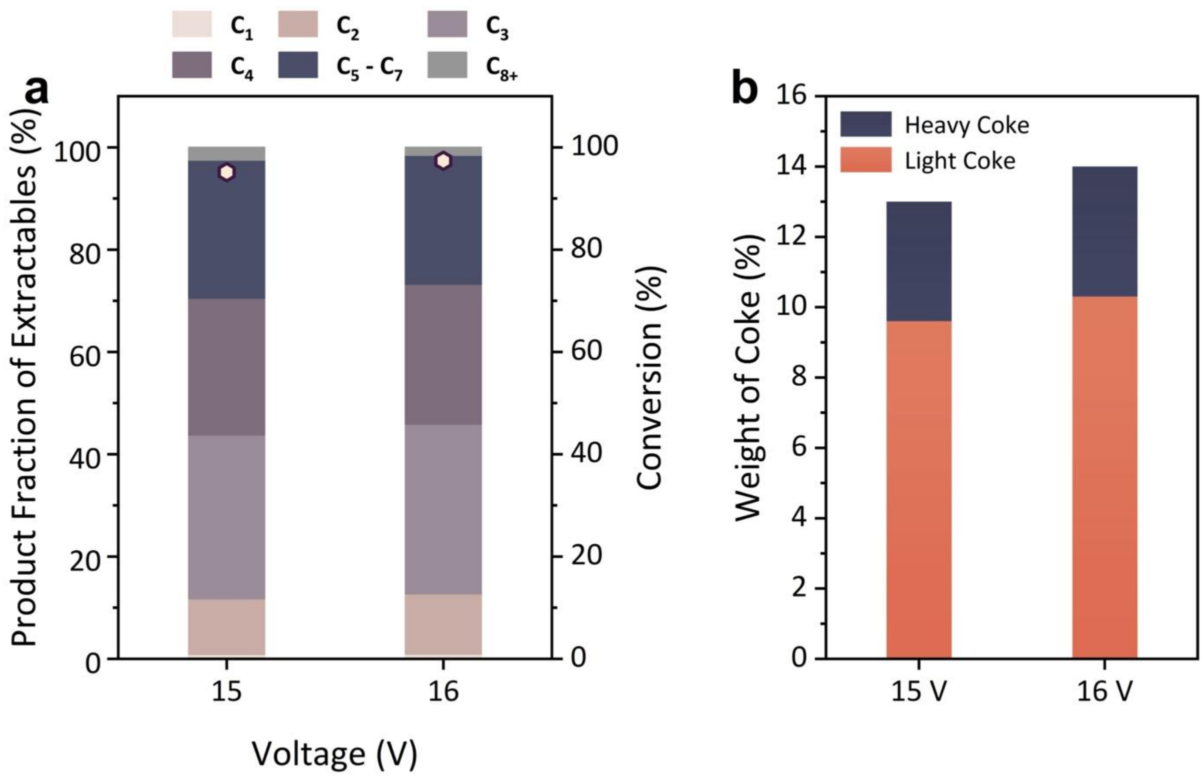


**Supplementary Figure 13.** (a) Effect of DC voltage on conversion and selectivity for CJH of LDPE over H-ZSM-5 (reaction time = 10 s). (b) Weight % of coke obtained from TGA of spent catalysts from CJH of LDPE (reaction time = 10 s).

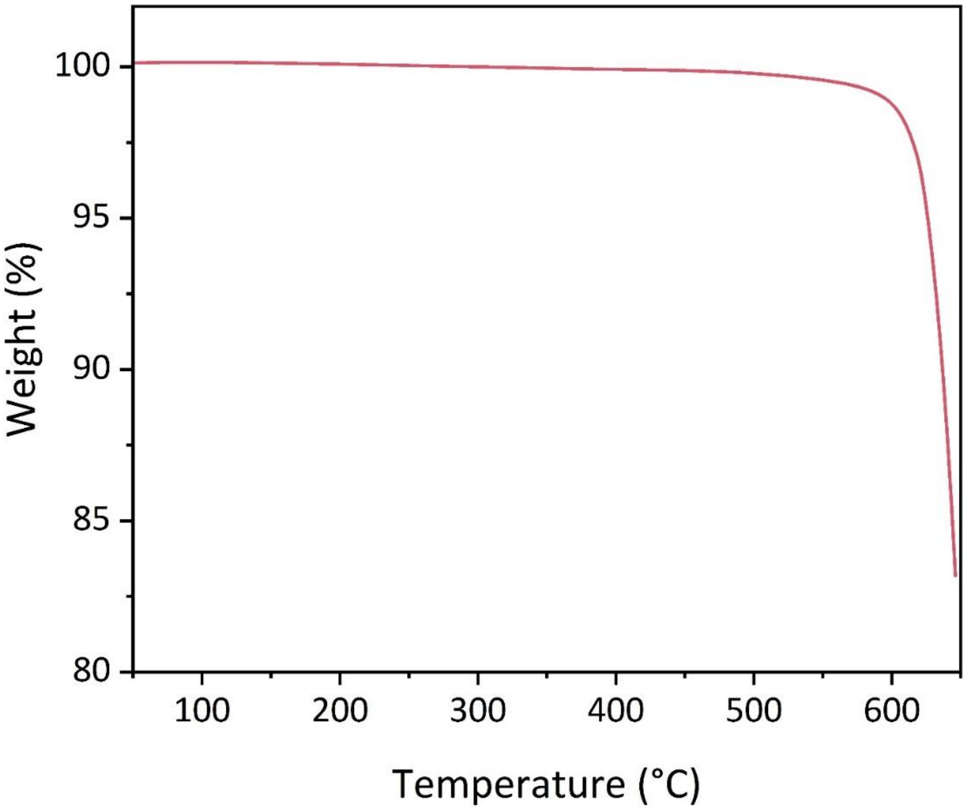


**Supplementary Figure 14.** TGA of fresh CFP (not coated with catalyst and polymer).

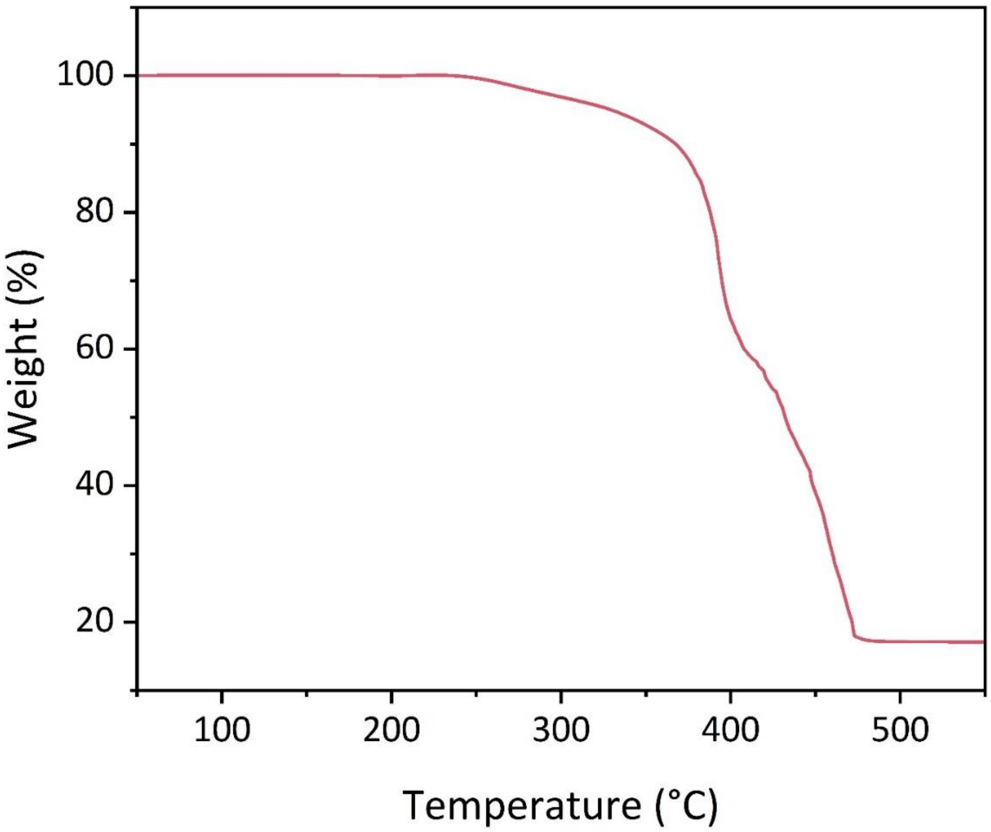

**Supplementary Figure 15.** TGA of HDPE grocery bag.

**Supplementary Table 1.** Maximum and average temperatures at different pulsing conditions measured using an IR camera.

| **Voltage** | **Operational Mode** | **Pulsing Parameters** | **$T_{max}$ (°C)** | **$T_{avg}$ (°C)** | **Carbon Balance (%)** |
|---|---|---|---|---|---|
| 32 V | RPH | 60 pulses, 50 ms | 540 | <476 | 86 |
| 36 V | RPH | 60 pulses, 50 ms | 630 | 495 | 84 |
| 42 V | RPH | 60 pulses, 50 ms | 752 | 542 | 86 |
| 42 V | RPH | 10 pulses, 50 ms | 730 | 510 | 83 |
| 20 V | CJH | 1 pulse, 500 ms | 495 | <475 | 94 |
| 23 V | CJH | 1 pulse, 500 ms | 565 | 505 | 92 |
| 25 V | CJH | 1 pulse, 500 ms | 648 | 550 | 84 |
| 26 V | CJH | 1 pulse, 500 ms | 748 | 625 | 81 |
| 27 V | CJH | 1 pulse, 500 ms | 815 | 670 | 84 |
| 31 V | CJH | 1 pulse, 500 ms | 1030 | >700 | 83 |
| 42 V | RPH | 5 pulses, 50 ms, 0.25 Hz | <475 | <475 | 98 |
| 42 V | RPH | 5 pulses, 50 ms, 0.5 Hz | 490 | <475 | 88 |
| 59.4 V | RPH | 10 pulses, 25 ms, 1 Hz | 775 | 518 | 91 |
| 59.4 V | RPH | 5 pulses, 25 ms, 1 Hz | 729 | 485 | 84 |
| 21 V | RPH | 10 pulses, 200 ms, 1 Hz | 690 | 497 | 89 |
| 21 V | RPH | 5 pulses, 200 ms, 1 Hz | 544 | <476 | 94 |
| 10.5 V | RPH | 5 pulses, 800 ms, 1 Hz | <476 | <476 | 95 |
| 15 V | CJH | 1 pulse, 10 s | 640 | 691 | 80 |
| 16 V | CJH | 1 pulse, 10 s | 744 | 810 | 92 |
| 53 V | RPH | 4 pulses, 50 ms, 0.5 Hz | 720 | 490 | 81 |
| 65 V | RPH | 2 pulses, 50 ms, 0.25 Hz | 716 | 485 | 85 |

*Several cases are labeled as <476 due to the inability of the IR camera to detect below 476 °C.

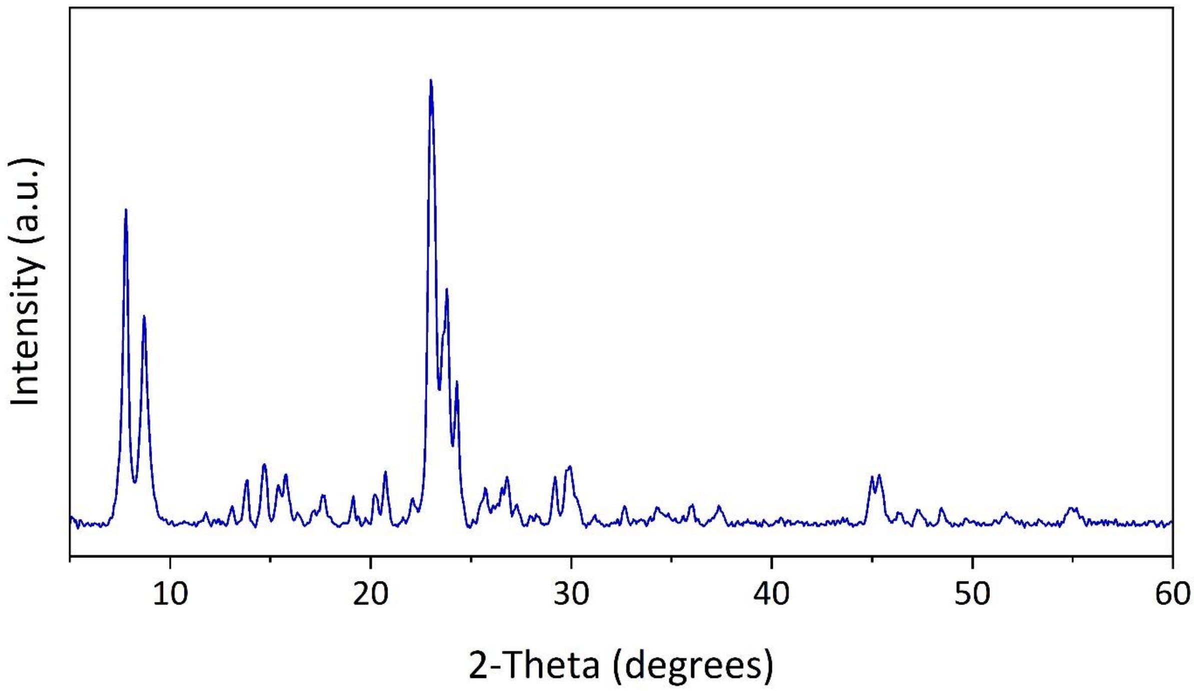


**Supplementary Figure 16.** X-ray diffraction pattern of H-ZSM-5 (30) catalyst.

**Supplementary Table 2.** Textural properties of different catalyst samples.

| Sample | BET Surface Area ($m^2$/g) | External Surface Area[a] ($m^2$/g) | $V_{micro}$[a] ($cm^3$/g) | Brønsted acid sites (μmol/g)[b] | Lewis acid sites (μmol/g)[b] | Elemental Loading (%) |
|---|---|---|---|---|---|---|
| **H-ZSM-5 (30)** | 250 | 88 | 0.145 | 338 | 32 | 5.88 |

[a] – obtained from t-plot results; [b] – measured using FTIR of adsorbed pyridine; [#] – Spent catalyst after calcination in static air at 550 °C.

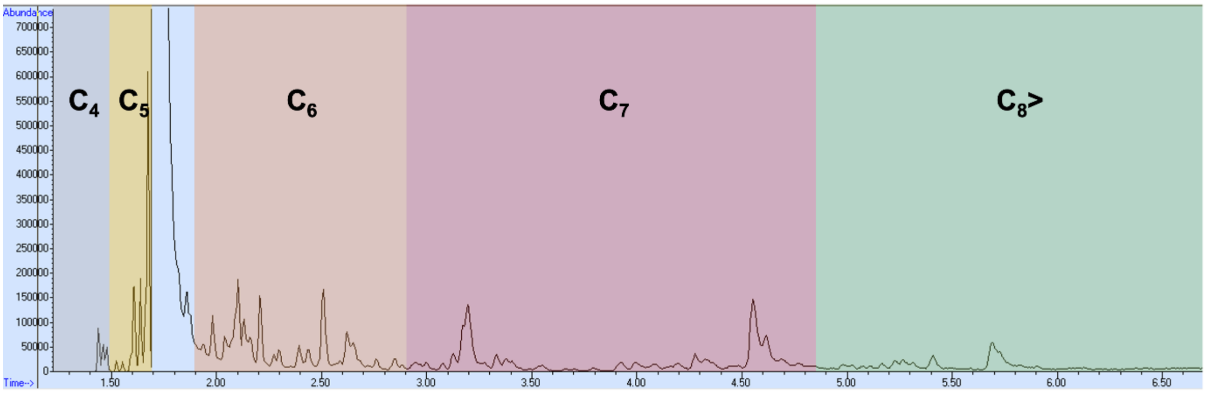


**Supplementary Figure 17.** GC-MS chromatogram of product mixture collected using a solvent trap to identify products >$C_3$.

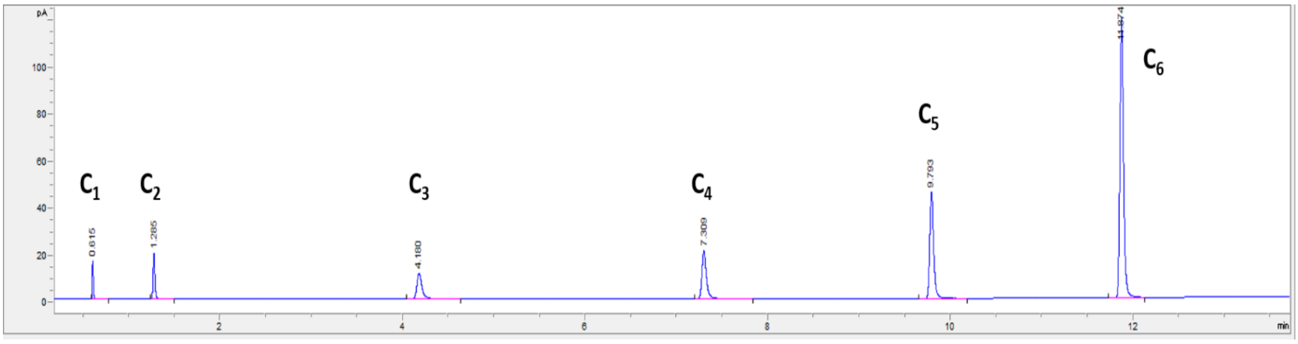


**Supplementary Figure 18.** GC chromatogram of gas calibration mixes.

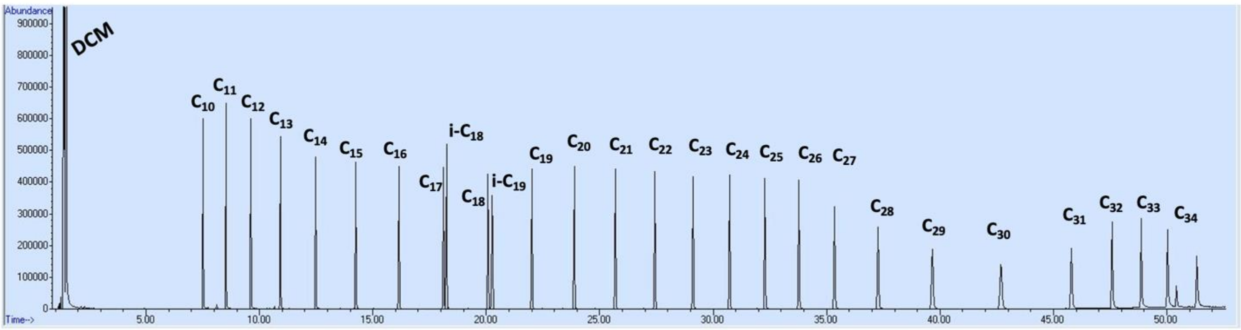


**Supplementary Figure 19.** GC-MS chromatogram of hydrocarbon-mix calibration standard 1.

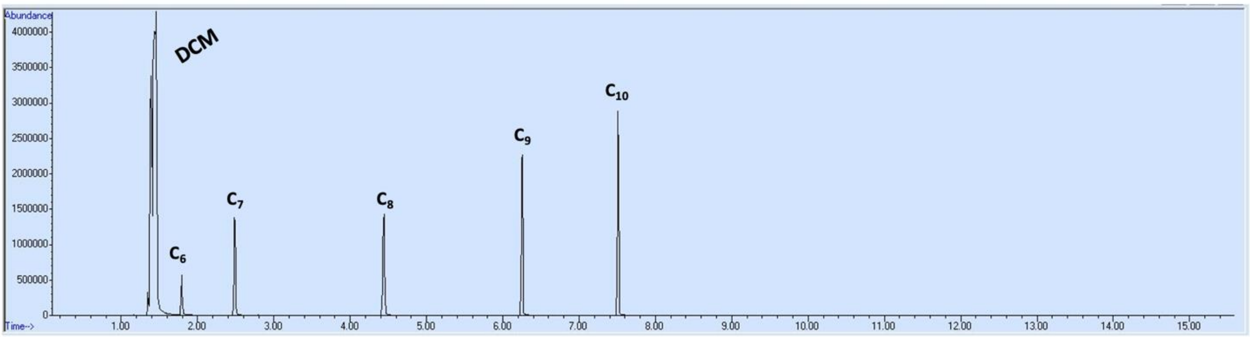


**Supplementary Figure 20.** GC-MS chromatogram of hydrocarbon-mix calibration standard 2.

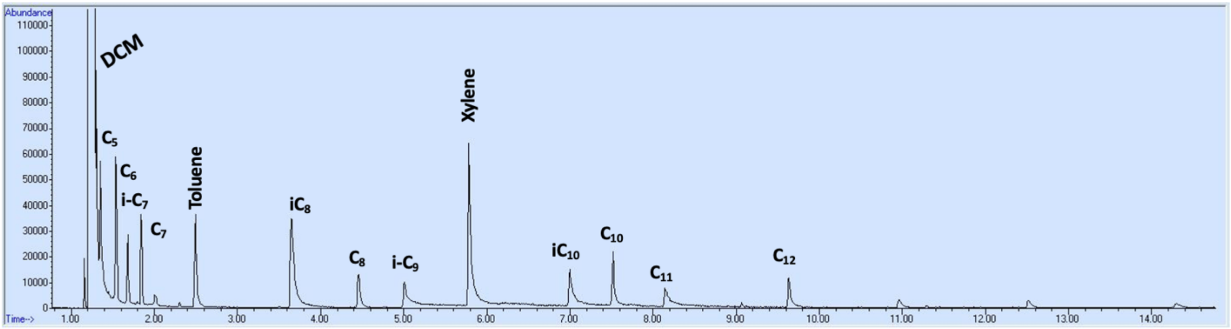


**Supplementary Figure 21.** GC-MS chromatogram of hydrocarbon-mix calibration standard 3.

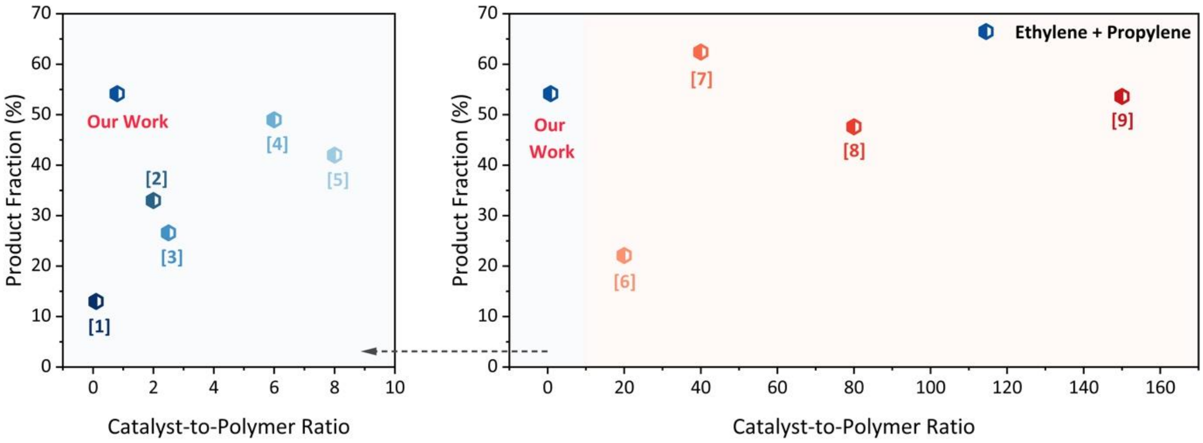


**Supplementary Figure 22.** Comparison of product fractions of ethylene plus propylene vs. catalyst-to-polymer ratio of this work to literature for the catalytic deconstruction of polyethylene[1-9]. Left panel is a zoom in at low catalyst to polymer ratios.

## Supplementary References


1 Selvam, E. *et al.* Plastic waste upgrade to olefins via mild slurry microwave pyrolysis over solid acids. *Chemical Engineering Journal* **454**, 140332, doi:https://doi.org/10.1016/j.cej.2022.140332 (2023).

2 Ali, S., Garforth, A. A., Harris, D. H., Rawlence, D. J. & Uemichi, Y. Polymer waste recycling over “used” catalysts. *Catalysis Today* **75**, 247-255, doi:https://doi.org/10.1016/S0920-5861(02)00076-7 (2002).

3 Sharratt, P. N., Lin, Y. H., Garforth, A. A. & Dwyer, J. Investigation of the Catalytic Pyrolysis of High-Density Polyethylene over a HZSM-5 Catalyst in a Laboratory Fluidized-Bed Reactor. *Industrial & Engineering Chemistry Research* **36**, 5118-5124, doi:10.1021/ie970348b (1997).

4 Zhou, N. *et al.* Catalytic pyrolysis of plastic wastes in a continuous microwave assisted pyrolysis system for fuel production. *Chemical Engineering Journal* **418**, 129412, doi:https://doi.org/10.1016/j.cej.2021.129412 (2021).

5 Artetxe, M. *et al.* Cracking of High Density Polyethylene Pyrolysis Waxes on HZSM-5 Catalysts of Different Acidity. *Industrial & Engineering Chemistry Research* **52**, 10637-10645, doi:10.1021/ie4014869 (2013).

6 Xue, Y., Johnston, P. & Bai, X. Effect of catalyst contact mode and gas atmosphere during catalytic pyrolysis of waste plastics. *Energy Conversion and Management* **142**, 441-451, doi:https://doi.org/10.1016/j.enconman.2017.03.071 (2017).

7 Eschenbacher, A. *et al.* Boron-Modified Mesoporous ZSM-5 for the Conversion of Pyrolysis Vapors from LDPE and Mixed Polyolefins: Maximizing the C2–C4 Olefin Yield with Minimal Carbon Footprint. *ACS Sustainable Chemistry & Engineering* **9**, 14618-14630, doi:10.1021/acssuschemeng.1c06098 (2021).

8 Eschenbacher, A. *et al.* Highly selective conversion of mixed polyolefins to valuable base chemicals using phosphorus-modified and steam-treated mesoporous HZSM-5 zeolite with minimal carbon footprint. *Applied Catalysis B: Environmental* **309**, 121251, doi:https://doi.org/10.1016/j.apcatb.2022.121251 (2022).

9 Eschenbacher, A., Varghese, R. J., Abbas-Abadi, M. S. & Van Geem, K. M. Maximizing light olefins and aromatics as high value base chemicals via single step catalytic conversion of plastic waste. *Chemical Engineering Journal* **428**, 132087, doi:10.1016/j.cej.2021.132087 (2022).
